# Deaths

**Published:** 1781-06

**Authors:** 


### DEATHS.

*July* 2, 1780.—At Halle in Saxony, in his 71ft year, David Samuel de Madai, M. D. phyfician to the orphan-houfe in that city. He was a native of Hungary, and celebrated for his erudition, and his knowledge of coins.

*April* 26, 1781.—At Amerfbury, Mr. Bloxham, apothecary.

*May* 20.—At Strabane, in Ireland, Mr. George Buchanan, apothecary.

*June.*—At Worcefter, aged 29 years, Mr. Thomas Blount Lovet, furgeon and apothecary.

Lately at Limerick in Ireland, John Barrett, M. D. an alderman of that city.

——At St. James's in Jamaica, aged 33 years, Mr. John Rankin, furgeon.

